# Hypertension in sub-Saharan Africa: bridging the gaps through implementation research and scientific innovation

**DOI:** 10.1093/eurheartj/ehag305

**Published:** 2026-06-05

**Authors:** Anthony O Etyang, Tomasz J Guzik, Pablo Perel

**Affiliations:** Department of Epidemiology and Demography, KEMRI-Wellcome Trust Research Programme, P.O. Box 230-80108, Kilifi, Kenya; Centre for Cardiovascular Sciences, Queens Medical Research Institute, University of Edinburgh, Edinburgh, UK; Department of Medicine, Jagiellonian University, Collegium Medicum, Krakow, Poland; African Research Universities Alliance ARUA & The Guild of European Research-intensive Universities, Rue du Trône 98, B - 1050 Brussels, Belgium; Department of Non-Communicable Disease Epidemiology, London School of Hygiene & Tropical Medicine, London, UK

Hypertension is the leading global risk factor for mortality, accounting for over 10 million deaths annually, yet its control remains the clearest expression of global cardiovascular inequity. Nowhere is this more visible than in Sub-Saharan Africa, where fewer than one in 10 individuals with hypertension achieve target blood pressure levels in spite of over 130 million individuals living with hypertension, a number projected to rise to 216 million by 2030.^1^ As the continent undergoes rapid urbanization, climate stress, and demographic transition, Sub-Saharan Africa is poised to become both the epicentre of the global hypertension epidemic and a proving ground for scalable cardiovascular solutions.

Advancing hypertension control in Sub-Saharan Africa rests on three pillars: first, global cardiovascular equity that addresses structural determinants and health system fragmentation; second, implementation and systems transformation that scales evidence-based interventions through digital innovation and task-sharing models; and third, innovation and precision prevention in African populations that leverages genomic diversity, unique biological risk factors and environmental exposures to advance global cardiovascular science^2^

## What we know

### The growing burden of hypertension and its determinants

Hypertension has emerged as the most prevalent risk factor for cardiovascular morbidity and mortality in Sub-Saharan Africa. According to World Health Organization estimates, hypertension is associated with over 700 000 deaths annually in the region, contributing significantly to stroke, heart failure, kidney disease, and premature mortality.^1^ Rapid urbanization, climate-driven heat exposure, air pollution, conflict and migration, as well as changing food systems now interact to create what may be termed a ‘global syndemic’—a convergence of biological, social, and environmental drivers amplifying cardiovascular risk.^2–4^ These transitions, occurring within fragile health systems, compound the challenge of managing the disease effectively.^4^

### Awareness and control rates are critically low

Hypertension control in Sub-Saharan Africa reflects the systems challenges across the care continuum, awareness, diagnosis, treatment, and sustained control, each constrained by fragmented infrastructure and economic vulnerability.^4^ However, recent WHO HEARTS control initiatives in sub-Saharan Africa and elsewhere^5^ demonstrate promising progress across several of these domains.

**Figure 1.**
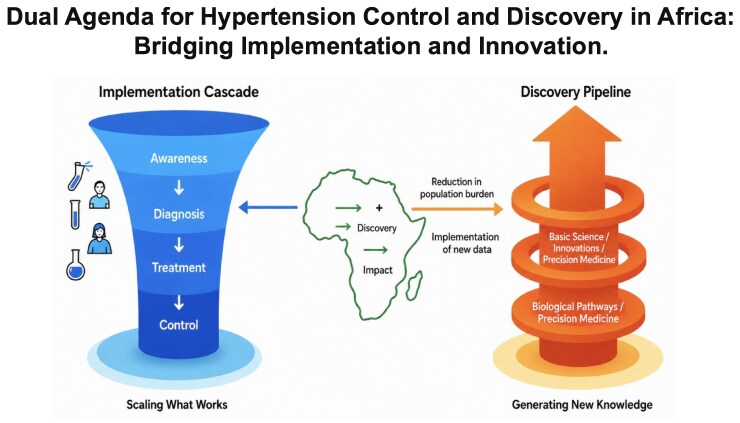
Dual agenda for hypertension control and discovery in Africa: bridging implementation and innovation

Stepping back, we can conceptualize (*Figure 1*) a parallel system-level cascade: from individual engagement to provider capacity, to the systemic enablers or barriers that shape outcomes.

### Multilevel barriers, solutions, and system transformation

**Individual level**: While hypertension remains asymptomatic until complications arise, innovative behavioural interventions, including text messaging, gamified adherence platforms, and community-based health education might be successful in addressing low health literacy and cultural barriers. Mobile health platforms are reducing distance and cost barriers while integrating traditional medicine approaches into comprehensive care models.^6^

**Provider level**: Many providers have limited training in non-communicable disease management. This is compounded by a severe shortage of health workers. Overburdened clinicians often lack time, tools, or support to deliver sustained care. Task-sharing models, use of point of care diagnostic tools and AI-enabled decision-support tools that improve diagnostic accuracy and enable non-physician providers to deliver effective hypertension care can expand provider capacity.

**System level**: Healthcare systems are characterized by fragmented care pathways, weak referral networks, poor information systems, limited insurance coverage, and frequent stockouts of antihypertensive medicines.^7^ Regional pooled procurement systems, digital supply chains that prevent stockouts, and integrated health information systems that support continuity of care are needed. Regional insurance mechanisms and innovative financing models would improve access to essential medicines.

In addition, broader structural determinants, including poverty, gender inequities, displacement due to conflict and climate-related problems, and digital infrastructure gaps, exert powerful influences on hypertension control yet remain outside most current models of care. Addressing these macro-determinants requires coordinated policy action across sectors.

## Promising models exist

No silver bullet exists, although a new generation of digital and implementation innovations are emerging in Sub-Saharan Africa. Community-based screening and task-sharing with nurses, community health workers, or trusted local leaders can improve diagnosis and management. mHealth platforms that support blood pressure monitoring and adherence are gaining traction. Single pill combination therapies simplify treatment and improve adherence. Integrating hypertension services into HIV programmes offers another opportunity, leveraging existing infrastructure and patient trust.^8^

Bridging the gap between evidence and widespread practice requires more implementation research. Questions remain on how best to adapt, adopt, implement, and scale these models. Task-sharing strategies are promising but must be evaluated for sustainability in real-world systems. mHealth tools require testing for cost-effectiveness, scalability, and integration. Financing models to ensure affordability, cultural acceptability, and workforce capacity also demand further exploration.

## What we don’t know

### Epidemiology: data gaps hinder planning

Despite the large burden of hypertension, most countries in Sub-Saharan Africa lack recent, nationally representative data on prevalence, treatment, and control. Longitudinal outcome data are also sparse. This hampers both individual risk stratification and population-level planning. Risk scores, key in clinical decision-making for people with cardiovascular risk factors, are largely developed in high-income countries and have not been validated in Sub-Saharan African contexts. Prognosis is not only critical for guiding care but also for informing health system planning and advocacy. Point-of-care diagnostics may support this, but require adaptation and study in local settings^9^

### Risk factors: context-specific drivers underexplored

Much of what we know about hypertension risk is derived from studies in high-income settings. Sub-Saharan Africa’s unique exposures, including chronic heat stress, early-life undernutrition, and infectious diseases like HIV and malaria, may alter risk and disease course.^10^ In addition, the region’s immense genetic diversity may lead to population-specific susceptibilities and drug responses. The prevalence and prognostic relevance of hypertension phenotypes, including masked hypertension, nocturnal hypertension, and salt sensitivity, remain underexplored in Sub-Saharan African populations but may be key to understanding population-specific risk patterns. Discovery research remains vital—to improve our understanding of mechanisms, risk stratification, and population-specific responses to treatment. These insights will not only inform precision prevention locally but also provide models of ‘reverse innovation’ for global cardiovascular medicine.

## Conclusion and call to action

Hypertension in Sub-Saharan Africa is a growing epidemic that demands urgent and coordinated action. While policy momentum for hypertension is increasing, with the recent Fourth High-level Meeting of the United Nations General Assembly on the prevention and control of NCDs and the promotion of mental health and wellbeing; in Sub-Saharan Africa this will require targeted investment in awareness, data systems, health workforce capacity, and access to essential medicines. Policymakers, researchers, and health providers must collaborate to close knowledge gaps, scale effective interventions, and embed hypertension control within broader health system strengthening. Improving hypertension control will also require a dual research agenda: advancing implementation science to scale proven interventions and investing in discovery research to better understand regional risk factors, disease patterns, and prognostic tools tailored to Sub-Saharan African populations. If this is achieved, Sub-Saharan Africa will emerge as a leader in cardiovascular innovation-a continent that transforms its burden into breakthrough solutions for global health.
